# Differences in the molecular profile of endometrial cancers from British White and British South Asian women

**DOI:** 10.1371/journal.pone.0233900

**Published:** 2020-06-10

**Authors:** Konstantinos Polymeros, David S. Guttery, Roger Hew, Rachael Bishop, Elizabeth Stannard, Salvador Macip, Paul Symonds, Esther L. Moss

**Affiliations:** 1 Leicester Cancer Research Centre, University of Leicester, Leicester, England, United Kingdom; 2 Department of Molecular and Cell Biology, University of Leicester, Leicester, England, United Kingdom; 3 Department of Pathology, University Hospitals of Leicester, Leicester, England, United Kingdom; 4 Department of Gynaecological Oncology, University Hospitals of Leicester NHS Trust, Leicester General Hospital, Leicester, England, United Kingdom; Sapporo Ika Daigaku, JAPAN

## Abstract

**Objectives:**

To identify differences in the mutational profile of endometrial tumours between British White (BW) and South Asian (BSA) women.

**Methods:**

We analysed primary tumours from matched cohorts of British White (BW) and British South Asian (BSA) women resident in Leicestershire diagnosed with EC. Next Generation Sequencing was performed to investigate mutational differences in a panel of 10 genes previously identified as being commonly mutated in EC. The presence of somatic Mismatch Repair (MMR) gene deficiencies was determined by immunohistochemistry.

**Results:**

In total, 57 tumours (27 BSA and 30 BW) were sequenced. There was no significant difference in the overall mutation frequency of the 10 genes analysed; however, numerous differences were observed between the groups. There was a positive association between *PIK3CA* and *PTEN* mutations in the BSA group, with 78% of *PIK3CA*-mutant tumours harbouring a *PTEN* mutation, whereas only 11% of *PIK3CA* wild-type (wt) tumours were *PTEN* mutant positive (p = 0.0012). In BW women, 90% of *ARID1A* mutant tumours had co-existent PI3K pathway mutations versus 50% of wild-type (wt) *ARID1A* patients (p = 0.0485). This trend was not significant in the BSA group (p = 0.66). The age at diagnosis was significantly higher in the BW group with a somatic MMR gene deficiency compared to those with no deficiency (72.8 years versus 59.6 years, p = 0.007), whereas this difference was not seen in the BSA group (64 years versus 60 years, p = 0.37).

**Conclusion:**

We have identified differences in the mutational profile of primary EC tumours from BW and BSA women. Further research is needed to confirm these findings and to explore their potential implications for early detection, treatment response and prognosis.

## Introduction

Internationally endometrial cancer (EC) is the second most common gynaecological malignancy with an estimated 382,069 new cases and 89,929 deaths worldwide in 2018 [1]. The incidence of endometrial cancer (EC) is typically higher in high-income countries, as compared to low-income countries however, the picture is changing with India in particular having the highest annual increase in EC incidence internationally between 2005 and 2007, a rise of 13% [2].

The issue of investigating ancestry and genetics in research is challenging due to the concept of race being confused or influenced by the interaction of environment and culture, as well as heterogeneity within populations [3]. Although it has been proposed that the use of race as a surrogate marker for measurable genetic differences should be avoided [4], it is acknowledged that there is utility when investigating the interplay of genes and environmental factors [5] and with a “biological correlate relevant to the disease” [6]. Much research has already been conducted in to the racial/ethnic differences in EC, predominantly from the USA, focusing on Black or African American (BoAA) and Caucasian women. A lower incidence of EC in BoAA women has been reported, as compared to Caucasian women [7–9], however, with a significantly worse prognosis [10]. Although, inequality in healthcare has been proposed as one reason for this difference it does not explain why BoAA women have greater propensity for developing serous subtypes as compared to other racial groups. The TCGA database has been utilised to look into this further and distinct molecular groupings have been identified in the EC tumours from BoAA and Caucasian women [11]. This supports the view that the clinical differences seen between BoAA and Caucasian women are due to underlying genetic differences, with a higher rate of *TP53* mutations and *ERBB2* amplification in tumours from BoAA women compared to tumours from Caucasian women [12,13], whereas the opposite is true for the frequency of *PTEN* mutations [14].

Limited evidence regarding the mutational landscape of EC exists for other geographic populations, with one of the least studied groups being Asian women. The categorisation of ‘Asian’ heritage in the medical literature is fraught with challenges and misconceptions, with Asia being the descriptive term for a large geographical area composed of many different cultural and environmental situations, which may share very few similarities. Therefore using the collective term Asian for women for the purposes of analysis could give misleading results and emphasises the need to be highly descriptive and avoid broad categories [15]. It could help to explain reported differences between data from the USA where the incidence of EC in Asian residents was found to be 40% less compared to the Caucasian population [8], whereas a UK study comparing White British with South Asian women living in the UK showed no difference (incidence rate ratio 0.90 vs 1, CI 0.81–1.01) [16]. One finding consistently reported however, is that the age at diagnosis is significantly lower in Asians as compared to Caucasian populations [17,18].

The aim of our study was to address this lack of evidence by investigating the mutational profile of genes commonly associated in the pathogenesis of EC in primary tumour specimens from women from two groups resident in Leicestershire: British White (BW) and Asian/Asian British–Indian/Pakistani, which we will refer to collectively as British South Asian (BSA). BSA is an accepted term that refers to a person whose ancestry originates in the Indian subcontinent, and who identifies with, or is identified with, their host country [19].

## Materials and methods

Ethical approval for the study was granted by Yorkshire and The Humber—Bradford Leeds Research Ethics Committee (REC reference number: 15/YH/0510). Women diagnosed with EC at the University Hospitals of Leicester between February 2016 and February 2018 were recruited. Informed written consent was obtained prior to participation and all underwent primary surgery, including hysterectomy. Participants’ ethnicity was self-designated using the National Office of Statistics for the decennial UK National Census classification.

Sixty of the patients recruited (30 BW and 30 BSA) who had confirmed histological diagnosis of EC were selected after being matched in terms of risk for adjuvant therapy using the ESGO-ESMO-ESTRO classification [20]. Based on this consensus document, patients with EC were categorised based on their risk of developing a recurrence to low, intermediate, high-intermediate, high, advanced and metastatic groups using FIGO stage, grade, histology, myometrial invasion, lymphovascular space involvement and residual disease following surgery. In total, 15 ‘low-risk’ and 15 intermediate/high-risk tumours were analysed from both the BW and BSA groups.

### DNA extraction

Formalin-Fixed, Paraffin Embedded (FFPE) blocks of the primary tumour were obtained. Following Haematoxylin-Eosin staining (H&E), a Consultant Pathologist marked a high density area of tumour and a 1.5mm core biopsy was performed. DNA was extracted using the QIAamp Generead DNA FFPE Tissue Kit (Qiagen) as per manufacturer’s instructions.

### Targeted Next Generation Sequencing (tNGS)

The Ion AmpliSeq designer software (https://www.ampliseq.com) was used to generate a custom 190 amplicon Ion AmpliSeq panel for analysis of hotspot regions in 10 genes commonly mutated in EC (*ARID1A*, *CTNNB1*, *FBXW7*, *KRAS*, *PIK3CA*, *PIK3R1*, *POLE*, *PTEN*, *PPP2R1A*, *TP53*) based on publicly available databases (https://portal.gdc.cancer.gov/) [21]. The panel was designed to contain two pools, against FFPE tissue, with amplicons ranging from 125–175 bp. Initial library preparation was performed using 20 ng of FFPE DNA. Samples were sequenced on a 318 chip using the Ion PGM Sequencer according to manufacturers’ instructions. Sequencing data was accessed and analysed through the Torrent Suite v5.6.0. All mutations with a quality score below 100 were omitted and all variants detected in the first or last 10 bases of an amplicon were omitted as likely mispriming events [22]. ANNOVAR [23] was used to annotate all mutations with refGene ID, functional consequence (e.g., non-synonymous), and functional predictions using SIFT [24], Polyphen-2 [25] and MutationTaster [26]. Known germline variants without pathological consequence were omitted from the analysis. All variants detected were also manually confirmed across all samples using the Integrated Genomics Viewer 2.3 [27,28]. The mutant allele frequency (termed AF) was calculated as the proportion of total reads at a site, which contained the variant allele (e.g. if you have 200 reads in total and 8 of them have the variant, then the AF is (8/200) x100 = 4%). TIER 1 and TIER2 drivers were identified using the cancer genome interpreter for SNVs, INDELs, and SCNA, with potential treatments analysed using the web-based analysis function at https://www.cancergenomeinterpreter.org.

### Immunohistochemistry

Immunohistochemistry was conducted using a Dako Autostainer in order to determine the presence of a somatic mutation within the four mismatch repair (MMR) genes associated with EC (*MSH2*, *MSH6*, *MLH1* and *PMS2*). The 5μM sections were incubated with the following primary antibodies; Monoclonal Mouse MLH1 (clone ES05), Monoclonal Mouse MSH2 (clone FE11), Monoclonal Rabbit MSH6 (clone EP49) and Monoclonal Rabbit PMS2 (clone EP51), all obtained from Dako (Santa Clara, CA 95051, USA). Antigen retrieval was performed in a Dako PTlink using a high pH target retrieval solution. Following a 30 minutes incubation with the primary antibodies, visualisation was carried out using the Dako enVision Flex + detection system for MSH6 and PMS2, with a mouse linker for MLH1 and MSH2. On-slide control tissue was used, along with internal control of background endometrium. Results were independently assessed by two pathologists.

### Statistical analysis

Chi-square and Fisher’s exact tests were used to compare categorical variables. Analysis was two-sided and differences were considered to be statistically significant at p<0.05. All analyses were performed using GraphPad Prism software, version 7.0 (GraphPad Software, Inc, CA, USA).

## Results

The mean body mass index (BMI) was lower in the BSA compared to the BW groups (32.5kg/m^2^ versus 37.8kg/m^2^, p = 0.0253), however the incidence of diabetes was more than double, 44% versus 20% respectively (p = 0.08) (Table 1). The BW patients with low-risk disease were significantly younger at diagnosis compared to intermediate- and high-risk patients (54.1 vs. 70.4 years old, p<0.0001) whereas this difference was not significant in the BSA group (59.4 vs. 63 years old, p = 0.39).

**Table 1:** Clinical and demographic data for the participants of the study whose tumours were sequenced. P values were calculated using Student’s t-test (unpaired) for age and BMI, Fisher’s exact test for histology and diabetes and Chi-Square test for stage and grade.

*Group*	*British White*	*British South Asian*	*P value*
*n*	*30*	*27*	
*Mean age (range)*	*62*.*3 (48–84)*	*61*.*2 (36–77)*	*0*.*7*
*Mean BMI (range)*	*37*.*8 (19–65)*	*32*.*5 (23–52*.*2)*	***0*.*0253***
*Histology*			*0*.*4*
*Endometrioid (%)*	*28 (93%)*	*23 (85%)*	
*Serous (%)*	*2 (7%)*	*4 (15%)*	
*Stage (FIGO)*			*0*.*88*
*1*	*25 (83%)*	*23 (85%)*	
*2*	*3 (10%)*	*3(10%)*	
*3*	*2 (7%)*	*1 (5%)*	
*Grade*			*0*.*37*
*1*	*20*	*13*	
*2*	*7*	*10*	
*3*	*3*	*4*	
*Diabetes (%)*			*0*.*08*
*Yes*	*6 (20%)*	*12 (44%)*	
*No*	*24 (80%)*	*15 (56%)*	

### Gene mutations

There were a total of 129 non-synonymous mutations in the 10 genes analysed, a mean of 2.3 per case (Table 2). For full details of each mutation per patient, see S1 Table. A few associations were seen across the whole cohort, in particular the presence of *TP53* mutations and older age at diagnosis (72.5 vs 60.5 years, p = 0.008). A similar association was seen between *PIK3CA* mutations and age (65.9 vs 59.3 years, p = 0.0245). No differences were identified in the incidence of non-synonymous mutations in these ten genes between the two groups although differences were seen in associations. In the BW cases, *ARID1A* mutations co-existed with PI3K pathway mutations (defined as mutations in either *PIK3CA*, *PIK3R1* or *PTEN*), as 90% (9/10) of *ARID1A* mutation carriers had a concurrent PI3K pathway mutation versus 50% (10/20) of wild-type (wt) *ARID1A* patients (p = 0.0485). This was not seen in the BSA cases (67% versus 52%, p = 0.66). Additional analysis was performed, including logistic regression, to adjust for BMI, grade and histology and this did not alter the result.

**Table 2:** Non-synonymous mutations in the 10 genes tested in all the tumours (n = 57). Coloured cells represent a mutated gene, the number indicating the number of non-synonymous mutations identified within the gene. MMR deficient tumours are indicated by coloured cells. The mutation frequency between the two groups (British White (BW) and British South Asian (BSA) were calculated using Fisher’s exact test.

Patient	Stage	Grade	Histology	ARID1A	CTNNB1	FBXW7	KRAS	PIK3CA	PIK3R1	POLE	PPP2R1A	PTEN	TP53	MMR
BW1	1A	1	Endometroid	1				2		1		2		
BW2	1A	1	Endometroid	1	1			1						
BW3	1A	1	Endometroid				1	1				1		
BW4	1A	3	Mixed (endometroid+serous)										1	
BW5	1B	2	Endometroid	1				2						
BW6	1A	1	Endometroid	1	1			1				1		
BW7	2	1	Endometroid			1	1	1						
BW8	1A	1	Endometroid			1								
BW9	1A	1	Endometroid	1					2	1		1		
BW10	1A	1	Endometroid	1								1		
BW11	1B	1	Endometroid				2	4		1		1		
BW12	1B	1	Endometroid											
BW13	1B	2	Endometroid				1		1			1		
BW14	3B	3	Endometroid	1				1		1			1	
BW15	1B	1	Endometroid					1				1		
BW16	1A	1	Endometrioid											
BW17	1A	1	Endometroid		1							1		
BW18	1B	1	Endometroid			1		1						
BW19	1A	1	Endometrioid	1										
BW20	2	2	Endometroid											
BW21	1A	1	Endometroid			1						2		
BW22	1A	1	Endometrioid											
BW23	3A	2	Endometrioid		1			2				1		
BW24	1A	1	Endometrioid											
BW25	2	1	Endometroid	1		1		2						
BW26	1B	2	Endometroid											
BW27	1A	2	Endometrioid			1						1		
BW28	1B	2	Endometrioid											
BW29	1A	3	Mixed (serous and clear cell)	1								1	1	
BW30	1A	1	Endometrioid											
BSA1	1A	2	Endometroid					1				2		
BSA2	1A	2	Endometroid		1	1								
BSA3	2	3	Serous					1					1	
BSA4	1A	1	Endometrioid					2				2		
BSA5	1A	2	Endometroid			1	1						1	
BSA6	1A	2	Endometroid						1					
BSA7	1B	1	Endometroid			1			2					
BSA8	1A	3	Clear cell											
BSA9	1B	2	Endometrioid		1									
BSA10	3C1	3	Serous										1	
BSA11	1A	1	Endometroid			1		1				1		
BSA12	1B	1	Endometroid											
BSA13	1A	1	Endometroid		1									
BSA14	1B	2	Endometroid		1			1				1		
BSA15	1B	2	Endometroid		1			1				1		
BSA16	1A	1	Endometroid	1	1	1			1	1				
BSA17	1A	3	Serous											
BSA18	1A	1	Endometrioid						1					
BSA19	1A	1	Endometrioid	1								1		
BSA20	2	2	Endometrioid	1							1			
BSA21	2	2	Endometrioid		2	2		2	2	1				
BSA22	1A	1	Endometrioid											
BSA23	1B	1	Endometrioid	1				1				1		
BSA24	1B	1	Endometrioid		1							1		
BSA25	1A	1	Endometrioid			1								
BSA26	1A	1	Endometrioid	2										
BSA27	1A	2	Endometrioid	1				1				1		
			**Group comparison p values**	0.39	0.19	0.75	0.35	0.78	0.24	0.67	0.47	0.59	1	0.54

In the BSA cases, somatic *CTNNB1* mutations tended to be younger at diagnosis (mean age 55.6 years compared to 63.5 for their wild type counterparts, p = 0.077), a finding not seen in the BW cohort (63.3 years for somatic *CTNNB1* mutation carriers compared to 62.1 for wt-*CTNNB1*, p = 0.85). 92% of the *CTNNB1* mutations (12/13) identified in both groups were found in exon 3, from codon 32 to 41 and all of them were missense. The majority of these alterations (67%, 8/12) affected Serine or Threonine residues (S33, S37 and T41). Ninety-three percent (13/14) of *FBXW7* mutations detected were missense as were all six *KRAS* mutations, with three due to a substitution of glutamine to histidine (Q61H), a mutation mainly reported in colorectal malignancies [29]. Interestingly, all four *KRAS*-mutant tumours in the BW group had co-existent PI3K pathway mutations versus only 38% (10/26) of wt-KRAS cases (p = 0.0365). Only one BSA case contained a *KRAS* mutation, and therefore no comparison was possible.

Across the whole cohort, there was a total of 30 *PIK3CA* mutations, all missense, of which 10 were located in the p85-binding domain of the gene, with 4 of the 10 being a gain-of-function R88Q mutation (all identified in BW group). Another common mutation was the E545 in the helical domain (13% of the total, n = 4, two BW and two BSA). There was a strong, positive association between *PIK3CA* and *PTEN* mutations in the BSA, but not BW, group in which 78% (7/9) of *PIK3CA*-mutant tumours were also carrying a *PTEN* mutation versus only 11% (2/18) for wt-*PIK3CA* tumours (p = 0.0012). Interestingly, *PIK3CA*-mutant patients in the BW group were significantly older at diagnosis (mean age 68.7 years versus 58 for wt-*PIK3CA*, p = 0.0077) but this was not seen in BSA group.

*PTEN* mutations in the BW group were more likely to localize in the phosphatase catalytic domain of the gene (53%, 8/15), whereas, in the BSA group the majority of *PTEN* mutations was observed in the C2 domain of the gene (64%, 7/11). These differences however did not reach significance (p = 0.168).

As BMI was the only variable significantly different between the BW and BSA groups further analysis was performed dividing the whole cohort into BMI <35kg/m^2^ and >35kg/m^2^. There was no significant difference in the number of mutations in the ten genes tested nor in the rates of MMR deficiency in these two groups. The only association identified was *PIK3CA* mutations co-existed more frequently with *PTEN* mutations in the <35kg/m^2^ BMI group (9/13, 69% of *PIK3CA*-mutant patients had co-existent *PTEN* mutations compared to 4/16, 25% of wt-*PIK3CA*, p = 0.027), which was not the case in the >35 kg/m^2^ BMI cohort (4/8, 50% versus 5/20, 25% respectively, p = 0.37).

### Tumour Mismatch Repair (MMR) protein deficiency

A quarter of tumours (14/57) exhibited a deficiency in at least one of the MMR genes (Table 3). There was no difference in the presence of mutations between the BW and BSA group (20% vs 30%, p = 0.54), with the most common mutation MLH1/PMS2 being identified in 4 cases (67%) in the BW group and 7 cases (88%) in the BSA group. MSH6 mutations were identified in one BW and one BSA case and a MSH2/MSH6 mutation was only seen in one BW case. The age at diagnosis was significantly higher in the BW group with a somatic MMR gene deficiency compared to those with no deficiency (72.8 years versus 59.6 years, p = 0.007), whereas this difference was not seen in the BSA group (64 years versus 60 years, p = 0.37).

**Table 3:** Patient demographics and MMR protein expression (MLH1/MSH2/MSH6/PMS2) on immunohistochemistry.

	MMR proficient n = 43	MMR deficient n = 14	p value
Grouping			0.54
BW	24 (56%)	6 (43%)	
BSA	19 (44%)	8 (57%)	
Mean age	59.8	67.8	0.014
Mean BMI	36.5	31.5	0.066
Diabetes status			0.51
Diabetic	15 (35%)	3 (21%)	
Non-diabetic	28 (65%)	11 (79%)	
Risk group^a^ (%)			0.55
Low	23 (53%)	6 (43%)	
Intermediate-High	20 (47%)	8 (57%)	
Grade (%)			0.16
1	26 (63%)	6 (43%)	
2	10 (23%)	7 (50%)	
3	5 (14%)	1 (7%)	

^a^ Risk groups for adjuvant therapy using the ESGO-ESMO-ESTRO classification (20)

Examining associations between MMR gene deficiencies and the non-synonymous mutations described previously showed that within the BSA group, there was a negative association between MMR deficiency and *CTNNB1* mutations as none of the eight *CTNNB1*-mutant patients was MMR-deficient versus 42% (8/19) of *CTNNB1*-wt tumours (p = 0.06). There was no such trend in the BW cohort as 25% (1/4) of patients with a somatic *CTNNB1* mutation was MMR deficient compared to 19% (5/26) of wt-*CTNNB1* patients (p = 1). A similar negative correlation was seen in the BSA group between MMR deficient tumours and *FBXW7* mutations (0/7, 0% of *FBXW7*-mutant patients had concomitant MMR deficiency versus 8/20, 40% on the *FBXW7*-wt patients, p = 0.068). The same was not seen in BW women as 17% (1/6) of *FBXW7*-mutant patients were MMR deficient compared to 21% (5/24) of wt-*FBXW7* women (p = 1).

## Discussion

In this study we have explored the mutational landscape of EC in a previously under investigated population. Leicester is an ethnically diverse region with 28.3% of the Leicester population identifying as ‘Indian’ in the 2011 National census (n = 93,335) [30]. By selecting a population living in the same region, being treated within the ‘free at the point of need’ NHS we have attempted to address the differences that may result from access to health care and geographical location, although it was not possible to determine the duration of residence in the UK of the women in the BSA group. The BSA population is of interest since the leading risk factors for EC are obesity and diabetes, both of which have been shown to have different thresholds in the BSA as compared to White Europeans [31]. BSA women have lower obesity cut-off points for the development of dysglycemia, 21.5kg/m^2^ compared to 30kg/m^2^ for White European women, which could impact on prevention strategies and identifying women at risk of EC. Unlike previous epidemiological studies on EC in Asian women, our BSA cohort was very homogeneous, the majority of women were of Guajarati Indian heritage [32], and despite the sample size being relatively small, it is larger than the paradigm shifting TCGA EC study [21], which included only 20 Asian patients, with no other geographical definitions.

The 10 genes of interest were selected based on their frequency in EC, for which there is a wealth of evidence [33], and their potential significance in guiding personalised therapy. We have shown a strong co-existence of *PIK3CA* and *PTEN* mutations in the BSA group, which was not seen in the BW group. The correlation between *PIK3CA* and *PTEN* has been reported previously in EC in a cohort of cases from Japan [34] and might have clinical implications since a large study of a variety of solid tumours (mainly colorectal and ovarian) showed that patients with *PIK3CA* and/or *PTEN* mutations had a significantly greater partial response to *PI3K/mTOR* inhibition [35] compared to wt-tumours of these two genes. Our results therefore raise the possibility of greater efficacy of these treatments in BSA, as compared to BW, populations.

Previous studies have shown an association between *ARID1A* and mutations in the *PI3K* pathway with *ARID1A* mutations independently increasing the downstream activation of *PI3K* pathway [36]. We have confirmed this finding in the BW but not in the BSA group, with BW cases being more likely to ‘need’ *ARID1A* suppression for *PI3K* pathway activation whereas cases in the BSA group were more likely to have an activated *PI3K* pathway due to co-existence of *PIK3CA* and *PTEN* mutations. Also, it has recently been shown that *ARID1A* mutations can render cancer cells sensitive to glutathione and glutamate-cysteine ligase synthetase catalytic subunit (GCLC) inhibitors [37], while they also increase the sensitivity of cancer cells to PI3K inhibitors [38]. It can therefore be proposed that combining PI3K with GCLC inhibitors could be an effective strategy for the 30% of endometrial cancers carrying an *ARID1A* mutation. Our data therefore support the need for a personalised approach to identify the presence of mutations in different populations before embarking on trials investigating novel drug combinations.

Studies in ovarian clear cell carcinoma (OCCC) have shown higher prevalence of the disease in East Asia compared to North America and Europe [39]. Since one of the most frequent mutations in OCCC is *ARID1A*, higher rates of such genetic alterations in the BSA cohort could be expected. On the contrary, we have shown no difference in the rate of *ARID1A* mutations between the two groups, which is consistent with our previous analysis of TCGA cohort (11). One possible explanation is that many epidemiological studies in OCCC were conducted in Japan, a very different phylogenetic and environmental population as compared to South Asia.

Identifying the MMR status is becoming increasingly important in EC since it guides screening for potential Lynch Syndrome cases but also drug therapy options, for example checkpoint inhibitors such as Pembrolizumab. Our reported rate of somatic MMR mutations (25%) is very similar to recent reports in the literature [40,41]. There was no difference in the prevalence of MMR deficiency in the two groups however, the higher age at diagnosis in the BW group supported the view that MMR testing should be universal rather than focused on the younger EC population. The association between older age at diagnosis and MMR deficiency has been previously shown in studies on colorectal cancer and confirms our findings [42,43]. Our study reports a trend associating obesity with decreased rates of MMR deficiency (mean BMI 36.5 for MMR proficient tumours compared to 31.5 for MMR deficient, p = 0.066), and although this trend is not significant, this association has been reported in both colorectal and endometrial cancer studies [42,44]. Reasons for this association are not clear, although previous studies in colorectal cancer had shown that hyperestrogenemia reduces the risk of MMR deficiency, and this may explain the higher incidence of MSI in thin and elderly patients [45]. With aging, estrogen receptors (ER) in colonic cells hypermethylate, leading to reduced ER expression, which subsequently increases the carcinogenic potential of the colonic cells [46]. It is unknown as to whether the same mechanism occurs in endometrial cells and could be the focus of future studies.

There was a negative association between *CTNNB1* mutations and MMR deficiency in our cohort, which supports evidence from previous larger study that showed that *CTNNB1* mutations were less frequent in MSI positive tumours [47], although the mechanism accounting for this negative correlation has yet to be elucidated.

The main limitation of our study was the relatively small number of cases analysed. The sample size meant that it was not possible to adjust for all clinico-pathological factors and it is possible that significant associations have not been identified due to lack of statistical power. The likelihood of the relatedness of the BSA, although possible, is unlikely given Leicester’s large South Asian population and a similar possibility could be raised for the BW population.

In summary, we have identified differences in the molecular profile of ten genes associated with EC in tumours from BW and BSA women. Further research is needed to confirm these findings in a larger population and to explore their potential implications for treatment response and prognosis.

## Supporting information

S1 Table (XLSX)
